# Enhancing peer teaching and psychological outcomes in medical education through structured formative assessment: a quasi-experimental study

**DOI:** 10.3389/fpsyg.2025.1710203

**Published:** 2025-12-19

**Authors:** Dawei Zhang, Kuibo Zhang, Junquan Chen, Binfang Shang, Zhongzhen Su, Qiang Wu, Lianjun Yang, Lili Xie, Hai Lv

**Affiliations:** 1 Department of Spine Surgery, Fifth Affiliated Hospital of Sun Yat-sen University, Zhuhai, China; 2 Department of Continuing Education, Fifth Affiliated Hospital of Sun Yat-sen University, Zhuhai, China; 3 Department of Ultrasound, Fifth Affiliated Hospital of Sun Yat-sen University, Zhuhai, China; 4 The First People's Hospital of Shaoguan City, Shaoguan, China

**Keywords:** peer-assisted learning, formative assessment, teaching self-efficacy, teaching anxiety, academic motivation, medical education

## Abstract

**Background:**

Peer-assisted learning (PAL) is an established approach in medical education, yet variability in teaching quality persists when peer tutors lack structured pedagogical support. This study examined whether integrating a structured formative assessment framework could enhance peer tutors’ teaching performance, teaching self-efficacy, and reduce teaching anxiety, as well as improve first-year students’ knowledge, academic motivation, and self-efficacy.

**Methods:**

A quasi-experimental, parallel- group study was conducted in three medical universities in Guangdong, China (2024–2025). Final-year medical students (*n* = 122) served as peer tutors and were allocated to an intervention (*n* = 61) or control groups (*n* = 61), each supervising 6–8 first-year students (total first-year students initially = 850; final analytic sample = 820) (intervention *n* = 411; control *n* = 409). The intervention integrated validated formative assessment tools—Mini-Clinical Evaluation Exercise (Mini-CEX), Direct Observation of Procedural Skills (DOPS), and Reflective Teaching Journals—alongside faculty feedback and self-reflection. Data were analyzed using linear mixed-effects models (LMM) accounting for student–tutor nesting. False discovery rate (FDR) correction was applied to control for multiple testing.

**Results:**

Significant group × time interactions favored the intervention group across all outcomes. Students taught by intervention-group tutors showed higher post-test knowledge (7.93 ± 0.31 vs. 5.28 ± 1.10; *F* = 54.9, *p* < 0.001, η^2^ = 0.18) and greater academic motivation and self-efficacy (both *p* < 0.001). Peer tutors demonstrated higher teaching self-efficacy (72.48 ± 5.73 vs. 69.13 ± 5.91; *F* = 22.3, *p* < 0.001, η^2^ = 0.15) and lower teaching anxiety (2.34 ± 0.33 vs. 2.65 ± 0.39; *F* = 17.1, *p* < 0.001, η^2^ = 0.14). Post-test performance measures (PES-TBL, Mini-CEX, DOPS) were consistently higher in the intervention group (all *p* < 0.001). Qualitative reflections revealed challenges in communication and confidence but documented progressive improvements in interaction and teaching clarity.

**Conclusion:**

This study provides preliminary evidence that integrating structured formative assessment into peer-assisted learning enhances tutors’ instructional competence, strengthens self-efficacy, and reduces teaching anxiety, while simultaneously improving students’ motivation and learning outcomes. Embedding formative assessment within PAL may represent a feasible and scalable strategy to improve teaching quality in medical education.

## 1 Introduction

Peer- assisted learning (PAL) has become an integral component of contemporary medical education, offering senior students opportunities to develop instructional competencies, leadership, and communication skills while supporting the academic development of junior learners (Brierley et al., 2022). Evidence across medical institutions demonstrates that PAL can enhance academic achievement, conceptual understanding, and learner engagement (Friel et al., 2018; Slabbert, 2024). PAL may also strengthen tutors’ professional confidence and reflective teaching practice, contributing to both pedagogical and psychological development (Pierce et al., 2024). In recent years, peer teaching has expanded within Asian medical schools, including China, where student-centered and interactive learning approaches have been increasingly incorporated into curricula (Yao et al., 2025).

Despite these documented benefits, concerns remain regarding the consistency and overall quality of peer-delivered instruction. Teaching effectiveness in PAL often depends on tutors’ prior experience and their ability to explain scientific content clearly and interactively (Herrmann-Werner et al., 2017). Because most peer tutors lack formal pedagogical preparation and structured support systems, the quality of peer-led sessions can vary considerably (Larios-Jones et al., 2024). These challenges have stimulated interest in strategies that can enhance peer tutors’ instructional competence and ensure that peer teaching achieves its intended educational value.

One promising strategy is the integration of structured formative assessment within peer tutoring processes. Formative assessment—which includes ongoing feedback, guided reflection, and constructive review—has been shown to improve teaching quality and learner outcomes in faculty- led educational settings (Morris et al., 2021; Irons and Elkington, 2021). However, its potential for strengthening peer tutors’ instructional skills has been insufficiently explored.

Although peer teaching has gained traction in Chinese medical universities and contributes to students’ autonomy and collaborative learning (Zhu et al., 2024; Wang et al., 2025), limited empirical evidence exists on whether structured formative assessment can enhance peer tutors’ teaching performance and improve students’ learning experiences in this context. Addressing this gap essential for developing evidence- informed frameworks that support high-quality peer instruction and sustained tutor development.

Guided by Bandura’s Social Cognitive Theory (SCT) and Self-Determination Theory (SDT) (Martin and Guerrero, 2020), the present study examines whether integrating structured formative assessment into PAL improves peer tutors’ teaching self-efficacy and reduces teaching anxiety, while also enhancing first-year students’ academic motivation and academic self-efficacy. SCT highlights the role of mastery experiences, feedback, and social modeling in shaping self-efficacy (Woreta et al., 2025), whereas SDT emphasizes the importance of competence and autonomy in fostering intrinsic motivation (Ryan and Deci, 2020). Accordingly, the intervention incorporated structured feedback and reflective components designed to promote tutors’ mastery and self-regulated teaching.

Although the advantages of PAL are well documented, little is known about how structured formative assessment influences teaching behaviors, psychological outcomes, and student learning within peer-teaching environments. This study addresses this gap by evaluating a structured formative assessment framework implemented across three medical universities in China. The findings aim to inform the development of scalable, evidence-based approaches that strengthen peer tutors’ instructional performance and enhance the quality of peer-assisted learning.

## 2 Methods and materials

### 2.1 Participants and study design

This study employed a quasi-experimental, parallel-group, mixed-methods design across three medical universities in Guangdong Province, China, during the second semester of the 2024–2025 academic year. Participants included final-year undergraduate medical students serving as peer tutors and first-year medical students who received peer teaching. All sessions took place in medical education units and clinical-skills classrooms at the participating universities.

Participants were recruited through convenience sampling in collaboration with teaching and learning offices at each institution. Although recruitment was voluntary and therefore non-random, peer tutors were allocated to intervention or control group using a computer-generated randomization list created by an independent researcher. Allocation concealment was ensured using sealed, opaque envelopes opened only after enrollment.

First-year students were also recruited by convenience sampling from the same courses or departments where peer-assisted teaching sessions were scheduled. Each peer tutor was paired with six to eight first-year students based on class schedule to minimize disruption to routine teaching. All first-year students followed the same group allocation as their respective tutor. To minimize evaluation bias, faculty raters conducting Mini-CEX and DOPS assessments were blinded to group allocation and completed a two-hour calibration workshop prior to data collection. PES-TBL ratings were student-reported and therefore could not be blinded.

Inclusion criteria for peer tutors were: final-year medical student, at least one year of prior peer-teaching experience, willingness to participate in structured formative assessment training, and availability to deliver at least six peer teaching sessions. Peer tutors were excluded if they withdrew voluntarily, missed required training, or failure to participate in the Mini-CEX and DOPS assessments.

First year medical students were eligible if they were enrolled in relevant course where peer teaching was occurred and provided written informed consent. Students were excluded if they missed more than one required session or withdrew voluntarily.

Sample size was calculated using G*Power 3.1 for a two-group repeated-measures design analyzed with linear mixed-effects models (LMM). Assuming a medium effect size (Cohen’s d = 0.5), 80% power, and *α* = 0.05, at least 51 tutors per group were required. Allowing 15% attrition, 60 tutors were recruited for each group. One additional volunteer was included, resulting in 61 tutors per group (*N* = 122). With each tutor supervising six to eight students, the estimated total number of first-year participants ranged from 720 to 960.

All participants were informed about the study purpose, procedures, potential benefits, and possible risks. Written informed consent was obtained prior to participation. The study followed the principles of the Declaration of Helsinki and was approved by the Ethics Committee of Sun Yat-sen University (Approval No. SYSU-20240304-28).

### 2.2 Assessments

All instruments demonstrated acceptable to excellent reliability in the current sample (Cronbach’s *α* range = 0.79–0.87; ICC range = 0.72–0.74; *κ* = 0.79). Validated Chinese versions were used for all self-report measures, and cross-cultural adaptation was ensured through pilot testing and expert review.

#### 2.2.1 Demographic questionnaire

A short demographic questionnaire was developed specifically for this study to collect baseline information, including age, gender, academic year, GPA, and prior peer teaching experience.

#### 2.2.2 Knowledge assessment

Knowledge of first-year students was assessed using a researcher-developed 10-item multiple-choice test administered before and after the peer-teaching program. The test was directly aligned with the course learning objectives and practical skills addressed during the peer teaching sessions. Each item had four response options with one correct answer (maximum score = 10). Content validity was ensured through independent review by two senior faculty experts in clinical medical education. Minor revisions were made following pilot testing with 10 non-participating students. Internal consistency in the current sample was acceptable (Cronbach’s *α* = 0.79).

#### 2.2.3 Peer Evaluation Scale for Team-Based Learning (PES-TBL)

The PES-TBL, a validated 16-item instrument, was used to assess the perceived teaching quality of peer tutors from the student perspective. It measures four domains: clarity of explanation, content organization, learner interaction and engagement, and professional attitude. Each item is rated on a 5-point Likert scale (1 = strongly disagree, 5 = strongly agree). The total score ranges from 16 to 80, with higher scores indicating stronger teaching performance. The Chinese version used in this study had previously demonstrated good validity and reliability in medical education settings (He et al., 2025). Internal consistency in the present sample was high (Cronbach’s *α* = 0.87).

#### 2.2.4 Mini Clinical Evaluation Exercise (Mini-CEX)

The Mini-CEX was used to assess peer tutors’ real-time teaching performance (Mortaz Hejri et al., 2017; Loerwald et al., 2018). It includes five key domains: teaching readiness, clarity of content presentation, use of practical examples, interaction with learners, and responsiveness to questions. Each item was rated on a 9-point scale (1 = unsatisfactory to 9 = excellent). Each tutor was observed at least twice by a senior faculty member. Inter-rater reliability, calculated using a two-way random-effects intraclass correlation coefficient (ICC), indicated good agreement (ICC = 0.72).

#### 2.2.5 Direct Observation of Procedural Skills (DOPS)

The DOPS tool was used to assess peer tutors during sessions that involved procedural or hands-on skills (Tang et al., 2025; Hu et al., 2025). The evaluation covered four dimensions: demonstration of technique, clarity of explanation, level of supervision and guidance provided, and the quality of feedback given to students. Each item was scored on a 5-point scale (1 = poor to 5 = excellent). Each tutor received at least two DOPS assessments. Faculty raters participated in a two-hour calibration session prior to data collection to ensure scoring consistency. Inter-rater reliability was acceptable (ICC = 0.74).

#### 2.2.6 Reflective Teaching Journal

To promote self-awareness and ongoing improvement, peer tutors completed a structured Reflective Teaching Journal after each teaching session (Xu et al., 2020). Tutors were instructed to briefly reflect on their strengths, identify aspects needing improvement, and outline specific goals for the next session. Additionally, tutors rated their own performance on a 3-point scale (1 = needs major improvement, 2 = acceptable, 3 = excellent). These journals were reviewed by faculty members who provided feedback to support growth. The format of the journal was adapted from existing educational literature (Xu et al., 2020; Ma et al., 2023). Data saturation was reached after iterative coding, and final themes were established through consensus meetings between the two coders. Qualitative entries were analyzed using conventional content analysis. Two independent coders reviewed and reconciled differences through discussion, achieving substantial inter-rater agreement (Cohen’s *κ* = 0.79). An inductive approach was adopted to allow categories to emerge directly from the data.

#### 2.2.7 Teaching self-efficacy

To measure peer tutors’ teaching self-efficacy, we used the short-form 20-item Teaching Self-Efficacy Scale developed and validated in a Chinese education context (Ma et al., 2023). This scale includes two subscales—Ethos (confidence in creating a positive, collaborative learning environment and engaging in professional development) and Teaching (tutors’ perceived ability to deliver clear explanations, facilitate student learning, and provide effective feedback during teaching sessions). Each item is rated on a 5-point Likert scale ranging from 1 = strongly disagree to 5 = strongly agree. The total score ranges from 20 to 100, with higher scores indicating greater perceived teaching self-efficacy. Construct validity of the scale was confirmed through exploratory and confirmatory factor analyses in the original study (Ma et al., 2023). In the current sample, internal consistency was high (Cronbach’s *α* = 0.87 for the total scale, and 0.79 and 0.81 for the Ethos and Teaching subscales, respectively).

#### 2.2.8 Teaching anxiety

Teaching anxiety was assessed using a Teaching Anxiety Scale (TAS), originally developed by Parsons (1973) and adapted by Chinese study (Liu and Yan, 2020). The scale included 33 items, covering common sources of teaching-related stress in higher education contexts. Responses were recorded on a 5-point Likert scale (1 = never to 5 = always). Total scores above 3 indicate high teaching anxiety, between 2 and 3 moderate anxiety, and below 2 low anxiety. The scale demonstrated good internal consistency in the present sample (Cronbach’s *α* = 0.83).

#### 2.2.9 Academic motivation

First-year students’ academic motivation was measured using the Academic Motivation Scale (AMS), grounded in Self-Determination Theory (Ten Cate et al., 2011). The validated Chinese version by Zhang et al. (2016) was used (Ten Cate et al., 2011). It comprises 28 items assessing intrinsic motivation, extrinsic motivation, and amotivation, rated on a 7-point Likert scale (1 = not at all true to 7 = completely true). Total and subscale scores were calculated by averaging the relevant items, with higher scores indicating greater levels of the respective motivation type (Hu and Luo, 2021). Internal consistency in this study was high, with Cronbach’s *α* = 0.80.

#### 2.2.10 Academic self-efficacy

Academic self-efficacy was assessed using the Chinese version of the Academic Self-Efficacy Scale (ASES-C), originally developed by McIlroy (2000) and later validated for Chinese university students (Zhao et al., 2024). This unidimensional 8-item scale is rated on a 7-point Likert scale (1 = not at all confident to 7 = extremely confident). Higher scores indicate stronger academic self-efficacy. The scale demonstrated good internal consistency in this study, with Cronbach’s α = 0.79.

### 2.3 Intervention

The peer tutors in the intervention group participated in a 2-h training workshop that taught the principles of effective peer teaching and how to use formative assessment tools such as Mini-CEX, DOPS, and the Reflective Teaching Journal. The workshop was facilitated by faculty members with experience in peer medical education.

During the 12-week peer teaching program, each peer tutor in both the intervention and control groups conducted six peer teaching sessions (each session lasting 60–90 min). The number and duration of sessions were standardized to ensure equal teaching exposure and comparability across groups. Session scheduling and oversight were coordinated by faculty coordinators at each university.

During this period, peer tutors of intervention group received direct observation and structured feedback from faculty using the Mini-CEX and DOPS tools at least twice for each section (theoretical and practical sessions). After each session, tutors were required to complete a Teaching Reflection journal, recording strengths, challenges, and plans for improvement for the next session.

In contrast, control group peer tutors conducted the same number and duration of peer teaching sessions with students, but received no formal training in formative assessment or structured feedback, and conducted their teaching activities according to standard peer teaching practice common at the participating universities.

To ensure methodological consistency across the three universities, all peer tutors followed an identical peer-teaching protocol. Teaching topics, learning objectives, session plans, lesson duration, teaching materials, and assessment rubrics (Mini-CEX, DOPS, and PES-TBL) were standardized and jointly developed by a committee of faculty representatives from all three institutions. Faculty observers also participated in a calibration workshop to ensure uniform scoring procedures. Therefore, the instructional content and assessment process were fully aligned across sites.

### 2.4 Statistical analyses

All data were analyzed using SPSS version 26.0 (IBM Corp., Armonk, NY). Descriptive statistics (mean, standard deviation, and frequency) were computed to summarize baseline characteristics. The normality of continuous variables was verified using the Shapiro–Wilk test and inspection of Q–Q plots prior to conducting parametric analyses. Moreover, pre-test differences were analyzed using independent-samples t-tests.

Because first-year students were nested within peer tutors (approximately 6–8 students per tutor), a multilevel analytic framework was applied to account for the hierarchical data structure and non-independence of observations. LMM with random intercepts for tutor ID were used to analyze primary outcomes, including knowledge scores, academic motivation, and academic self-efficacy for students, as well as teaching self-efficacy and teaching anxiety for peer tutors. The proportion of variance attributable to tutor clustering (ICC) was 0.13 for knowledge scores, 0.09 for academic motivation, and 0.11 for academic self-efficacy, supporting the use of LMMs with random intercepts for tutor ID. Fixed effects included group (intervention vs. control), time (pre vs. post), and their interaction (group × time), while baseline scores were entered as covariates where applicable. Model assumptions were checked by examining residual plots for normality, homoscedasticity, and influential outliers. Restricted maximum likelihood (REML) estimation was used, and model fit was evaluated using Akaike Information Criterion (AIC) and Bayesian Information Criterion (BIC). Effect sizes were calculated using partial eta-squared (η^2^) and interpreted according to Cohen’s benchmarks.

For outcomes without pre–post measures (e.g., PES-TBL, Mini-CEX, DOPS), independent t-tests were conducted at the tutor level, and findings were cross-validated using LMMs including tutor as a random effect to confirm robustness. Statistical significance was set at *p* < 0.05 (two-tailed).

Qualitative data derived from the reflective teaching journals were analyzed using conventional inductive content analysis to allow themes to emerge directly from the data rather than from a pre-existing framework. Two researchers independently conducted open coding, grouped similar codes into subcategories, and iteratively refined them into broader themes. The coding framework was discussed and finalized through consensus meetings. Data saturation was reached when no new categories emerged after reviewing approximately 90% of the journals. Inter- coder reliability was assessed using Cohen’s kappa (*κ* = 0.79), indicating substantial agreement. Final themes were confirmed through peer debriefing with an experienced qualitative researcher to enhance trustworthiness. Four overarching themes were identified: (1) difficulty managing group discussions, (2) communication barriers with first-year students, (3) low teaching confidence during early sessions, and (4) uncertainty about content accuracy.

To control for potential inflation of Type I error due to multiple testing, false discovery rate (Benjamini–Hochberg) correction was applied across the five primary outcome models; all main findings remained significant after FDR adjustment. Sensitivity analyses using Bonferroni correction produced the same pattern of results.

## 3 Results

A total of 122 peer tutors (61 in the intervention group and 61 in the control group) participated in the study, and all completed the intervention as scheduled without dropout. Each peer tutor supervised between six and eight first-year students during the 12 week program, resulting in 850 first-year students (425 per group). During the intervention, 14 first-year students in the intervention group and 16 in the control group withdrew due to absenteeism or voluntary withdrawal. Consequently, data from 411 intervention and 409 control students were retained for the final analysis.

The mean age of peer tutors was 23.9 ± 0.4 years and 55.73% were female (*n* = 68). Their mean GPA was 3.44 ± 0.15 on a 4.0 scale, and all had least 1 year of previous peer teaching experience. The mean age of first -year students was 18.91 ± 0.22 years, with 53.9% female (*n* = 442). There were no significant baseline differences between groups in demographic or outcome variables, indicating good initial comparability (Table 1).

**Table 1:** The baseline demographic characteristics of peer tutors and first-year students in the intervention and control groups.

Variable	Peer tutors			First-year students		
	All (*n* = 122)	Intervention (*n* = 61)	Control (*n* = 61)	All (*n* = 820)	Intervention (*n* = 411)	Control (*n* = 409)
Age, mean ± SD (years)	23.9 ± 0.4	24.1 ± 0.42	23.7 ± 0.38	18.91 ± 0.22	19.03 ± 0.23	18.79 ± 0.21
Female, *n* (%)	68 (55.73%)	33 (54.09%)	35 (57.37%)	442 (53.9%)	214 (52.06%)	228 (55.74%)
GPA (4.0 scale), mean ± SD	3.44 ± 0.15	3.36 ± 0.14	3.52 ± 0.16			
Teaching self-efficacy, mean ± SD	67.99 ± 6.4	68.05 ± 6.38	67.93 ± 6.42			
Teaching anxiety, mean ± SD	2.80 ± 0.41	2.82 ± 0.4	2.78 ± 0.41			
Academic motivation, mean ± SD				4.21 ± 0.6	4.23 ± 0.59	4.19 ± 0.612
Academic self-efficacy, mean ± SD				4.42 ± 0.51	4.41 ± 0.52	4.43 ± 0.5
Pre-test knowledge, mean ± SD				3.16 ± 0.20	3.20 ± 0.19	3.13 ± 0.21

Because students were nested within peer tutors (approximately 6–8 students per tutor), all inferential analyses were conducted using LMMs to account for the hierarchical data structure and non-independence of observations (Table 2). Each model included group (intervention vs. control), time (pre vs. post), and their interaction (group × time) as fixed effects, while tutor ID was included as a random intercept to account for within-tutor clustering.

**Table 2:** Linear mixed-effects model results comparing pre–post outcomes between intervention and control groups.

Variable	Post-test mean ± SD		F	*p*-value	η^2^	95% CI for Δ (Intervention − Control)	Effect size
	Intervention group	Control group					
Knowledge score	7.93 ± 0.31	5.28 ± 1.10	54.9	< 0.001	0.18	[2.32, 3.03]	Large
Teaching self-efficacy	72.48 ± 5.73	69.13 ± 5.91	22.3	< 0.001	0.15	[2.14, 4.66]	Large
Teaching anxiety	2.34 ± 0.33	2.65 ± 0.39	17.1	< 0.001	0.14	[−0.41, −0.14]	Large
Academic motivation	4.57 ± 0.54	4.27 ± 0.59	27.5	< 0.001	0.06	[0.23, 0.39]	Moderate
Academic self-efficacy	4.79 ± 0.47	4.53 ± 0.51	37.1	< 0.001	0.07	[0.18, 0.33]	Moderate

Means ± SD represent raw descriptive values; F = F-statistic from linear mixed-effects model; p = probability value (two-tailed); Partial η^2^ = effect size measure for explained variance, interpreted as small (≈0.01), medium (≈0.06), and large (≈0.14) following Cohen’s benchmarks. CI = confidence interval for group mean differences (Δ).

Significant group × time interaction effects were observed for all primary outcomes, indicating that the intervention group improved more over time than the control group. Specifically, knowledge scores showed a large improvement among students taught by intervention-group tutors (*F* = 54.9, *p* < 0.001, partial η^2^ = 0.18). Teaching self-efficacy among peer tutors also increased significantly compared with the control group (*F* = 22.3, *p* < 0.001, η^2^ = 0.15), while teaching anxiety decreased markedly (*F* = 17.1, *p* < 0.001, η^2^ = 0.14). Among first-year students, both academic motivation (*F* = 27.5, *p* < 0.001, η^2^ = 0.06) and academic self-efficacy (*F* = 37.1, *p* < 0.001, η^2^ = 0.07) increased significantly more in the intervention group than in the control group. Model diagnostics confirmed that residuals were normally distributed, variances were homogeneous, and no influential outliers were detected. Descriptive means (means ± SD) are reported in Table 2 for interpretability; inferential statistics (F, p, partial η^2^) are derived from linear mixed-effects models (LMM) adjusted for baseline scores and GPA.

For performance-based measures collected only at post-test, independent-samples t-tests were performed at the tutor level, and results were cross-validated using LMMs with tutor as a random effect (Table 3). Both analyses yielded consistent results, indicating significantly higher teaching performance among intervention-group tutors. The intervention group achieved higher mean scores on the Peer Evaluation Scale for Team-Based Learning (PES-TBL) (67.35 ± 5.77 vs. 59.32 ± 6.21; t (120) = 7.61, *p* < 0.001, Cohen’s d = 0.47), Mini-Clinical Evaluation Exercise (Mini-CEX) (7.76 ± 0.72 vs. 6.38 ± 0.88; t (120) = 8.15, *p* < 0.001, *d* = 0.42), and Direct Observation of Procedural Skills (DOPS) (4.45 ± 0.32 vs. 3.73 ± 0.41; t (120) = 8.13, *p* < 0.001, *d* = 0.82), corresponding to moderate-to-large effect sizes.

**Table 3:** Post-test comparisons for tutor performance indicators (independent-samples t-tests, confirmed with LMMs).

Variable	Intervention group (*n* = 61)	Control group (*n* = 61)	t (df)	*p*-value	Cohen’s d	Effect size interpretation
PES-TBL score	67.35 ± 5.77	59.32 ± 6.21	7.61	< 0.001	0.47	Moderate
Mini-CEX score	7.76 ± 0.72	6.38 ± 0.88	8.15	< 0.001	0.42	Moderate
DOPS score	4.45 ± 0.32	3.73 ± 0.41	8.13	< 0.001	0.82	Large

Values are presented as mean ± standard deviation. Independent samples t-tests were conducted to compare the intervention and control groups. Cohen’s d was calculated to indicate effect size.

Of the 372 reflective teaching journals collected from peer tutors, six were excluded due to incomplete or missing entries, leaving 366 journals for analysis. Based on tutors’ self-ratings, 56.5% of reflections were classified as acceptable, 21.2% as excellent, and 22.3% as requiring major improvement. Content analysis revealed four recurring themes: communication barriers with first-year students, challenges in managing group discussions, uncertainty about content accuracy, and low teaching confidence during early sessions. Tutors frequently identified goals related to improving interaction, time management, and clarity of explanations. Representative excerpts included statements such as: “At first, I was nervous about whether my explanations were clear enough, but after feedback, I realized the importance of checking students’ understanding during class,” and “Some students were quiet at first, and I found it difficult to engage them. Gradually, I learned to use questions to make them participate more.” These reflections suggest that structured formative assessment, combined with guided self-reflection, facilitated the development of tutors’ confidence, communication, and pedagogical adaptability throughout the intervention.

All primary results remained statistically significant after Benjamini–Hochberg FDR correction (adjusted *p* < 0.05); the same pattern was observed with Bonferroni correction, confirming robustness.

## 4 Discussion

This quasi-experimental study evaluated whether integrating structured formative assessment into peer-assisted learning enhances teaching quality among peer tutors and learning outcomes among first-year students. The observed improvements in first-year students’ knowledge scores and peer tutor’ teaching indicators (PES-TBL, Mini-CEX, and DOPS) provide preliminary evidence that formative assessment is a practical approach to improving the educational impact of peer tutoring, consistent with previous findings in health professions education (Herrmann-Werner et al., 2017; Sabale et al., 2022; Feng et al., 2024).

Structured formative feedback appeared to benefit both tutors and learners. Students taught by tutors receiving targeted feedback experienced clearer explanations, better-organized sessions, and more interactive instruction, which are known to enhance knowledge retention (Morris et al., 2021). Although modest gains occurred in the control group, the larger improvements in the intervention group underscore that structured observation and feedback enable tutors to refine delivery, adapt explanations, and address misunderstandings more effectively, aligning with constructivist and experiential learning frameworks (O’Connor and McCurtin, 2021).

Mechanisms driving teaching performance improvement include the use of Mini-CEX and DOPS, which facilitated structured observation and immediate feedback, promoting deliberate practice and competency development among novice educators (Lörwald et al., 2019; Lee and Mori, 2021; Embo et al., 2010). The PES-TBL scale provided clear performance criteria, reducing ambiguity and guiding tutors to follow recognized best practices (Robertson et al., 2025). Reflective journal analysis reinforced these findings: over half of reflections rated teaching as “acceptable,” with about one-fifth rated as “excellent,” accompanied by recurrent notes on communication and content clarity. This pattern demonstrates how guided reflection fosters metacognitive engagement and adaptability, core elements of reflective professional practice (Ratminingsih et al., 2017; Silver et al., 2023). Together, structured feedback and reflection appear to form a cycle that accelerates tutors’ professional growth and self-regulation (Zlabkova et al., 2024).

Formative assessment also yielded psychological and motivational benefits. Peer tutors in the intervention group reported higher teaching self-efficacy and lower teaching anxiety. According to Bandura’s social cognitive theory, formative feedback serves as a structured source of mastery experiences—the most powerful determinant of self-efficacy (Morris et al., 2021; Granziera and Perera, 2019; Lent, 2016). Constructive feedback following teaching attempts reinforced confidence and framed errors as growth opportunities, likely explaining reduced anxiety and creating a positive feedback loop between competence and performance (Jones et al., 2021; Patra et al., 2022).

First-year students in the intervention group demonstrated higher academic motivation and self-efficacy, which can be interpreted through Self-Determination Theory (SDT). The structured environment likely enhanced competence via clear instruction, autonomy through active engagement, and relatedness through tutors’ responsiveness and confidence (Ten Cate et al., 2011; Patra et al., 2022; Luarn et al., 2023). Consequently, improved teaching quality and psychological safety likely enhanced intrinsic motivation and belief in academic capability.

In summary, formative assessment in peer-assisted learning offers dual pedagogical and psychological benefits, reinforcing tutors’ instructional competence and confidence while simultaneously promoting learners’ motivation and self-efficacy. By bridging social-cognitive and self-determination perspectives, formative feedback fosters a self-reinforcing cycle of mastery, reduced anxiety, and sustained motivation for both tutors and learners.

Several limitations should be considered when interpreting these findings. First, the quasi-experimental design and convenience sampling restrict causal inference and may introduce selection bias, as students who volunteered to participate could have been more motivated or teaching-oriented than the general student population. Second, the relatively short intervention period did not allow for long-term follow-up, making it unclear whether improvements in teaching performance, motivation, or self-efficacy are sustained over time. Third, although reflective journals provided valuable qualitative insight, the absence of interviews or focus groups limited the depth of qualitative interpretation.

Additionally, a potential Hawthorne effect cannot be ruled out; tutors who knew they were being observed or receiving structured feedback may have temporarily altered their behavior, contributing to short-term performance gains. Finally, because the study was conducted in three universities within a single province in China, cultural and contextual factors may limit the generalizability of findings to other regions or educational systems. Future research would benefit from multi-center randomized designs, longer follow-up periods, and mixed-methods approaches incorporating interviews to further explore the mechanisms linking formative assessment, self-efficacy, and learning outcomes.

## 5 Conclusion

This quasi-experimental study indicates that integrating structured formative assessment into peer-assisted learning can enhance both teaching quality and student learning in undergraduate medical education. Peer tutors who received structured feedback through Mini-CEX, DOPS, and reflective journals demonstrated higher teaching performance, greater teaching self-efficacy, and lower teaching anxiety. Correspondingly, first-year students taught by these tutors achieved higher knowledge gains, stronger academic motivation, and improved academic self-efficacy. These findings suggest that formative assessment exerts a dual influence: it strengthens tutors’ instructional competence and confidence while fostering a more engaging and motivating learning environment for students. The consistent improvements across cognitive, behavioral, and psychological domains underscore the value of formative assessment as both an educational strategy and a professional development tool. Given its low cost and feasibility, this approach provides medical schools with a practical means to enhance the quality of peer teaching. Future research should explore the long-term sustainability of these effects and investigate the applicability of formative assessment models across different disciplines and cultural contexts.

## Data Availability

The raw data supporting the conclusions of this article will be made available by the authors, without undue reservation.
